# Sociodemographic differences in 10-year time trends of emotional and behavioural problems among adolescents attending secondary schools in Amsterdam, The Netherlands

**DOI:** 10.1007/s00787-018-1157-5

**Published:** 2018-04-26

**Authors:** Cornelia Leontine van Vuuren, Daan G. Uitenbroek, Marcel F. van der Wal, Mai J. M. Chinapaw

**Affiliations:** 1 Department of Epidemiology, Health Promotion and Healthcare Innovation, Public Health Service (GGD) Amsterdam, PO Box 2200, 1000 CE Amsterdam, The Netherlands; 2 Department of Public and Occupational Health, Amsterdam Public Health Research Institute, VU University Medical Center, Van der Boechorststraat 7, 1081 BT Amsterdam, The Netherlands

**Keywords:** Time trends, Adolescence, Mental health, Epidemiology, Sociodemographic factors

## Abstract

Societal change in western societies may impact emotional and behavioural problems of adolescents. Firm epidemiological evidence of changes in emotional and behavioural problems during the last decade is lacking. Insight into secular changes in emotional and behavioural problems among adolescents from various sociodemographic groups is crucial for adequate and targeted policy making. Therefore, the purpose of this study was to examine 10-year time trends in emotional and behavioural problems among adolescents, and potential differences in time trends between sociodemographic groups. Analyses were based on annually repeated cross-sectional data including 56,159 multi-ethnic students (13–14 years old) in the second year of various levels of secondary education in Amsterdam, The Netherlands, using the internationally validated Strengths and Difficulties Questionnaire. In general, emotional and behavioural problems increased over a 10-year time period (i.e., relative increase of total difficulties by 19%). This increase was mainly due to an increase in hyperactivity/inattention problems, while peer-relationship problems decreased. Time trends differed somewhat by sex: total difficulties and emotional problems increased in girls but remained fairly stable in boys. In Amsterdam, emotional and behavioural problems in adolescents seemingly increased over time, especially hyperactivity/inattention problems. Further research is needed to clarify the underlying causes. We cannot totally exclude potential confounders underlying our findings. Our findings can inform policies to target health programs at sociodemographic groups at increased risk.

**Electronic supplementary material:**

The online version of this article (10.1007/s00787-018-1157-5) contains supplementary material, which is available to authorized users.

## Introduction

Societal change in western societies may impact emotional and behavioural problems of adolescents. Adolescents appear to be more stressed, possibly due to factors such as the rise of social media and cyber bullying, the increasing proportion of single-parent families, greater emphasis on examinations and academic performance, and the ideal body image portrayed by the media [1–7].

Serious emotional and behavioural problems usually begin during adolescence and have implications for daily life, such as school attendance, ability to learn, substance use, violence, and social relations. Emotional and behavioural problems among adolescents tend to persist into adulthood. Addressing adolescents’ social–emotional needs is important for a healthy personal development, to prevent health and mental health problems later in life, and to improve social participation [6, 8–10]. The above-mentioned societal changes and the associated changes in emotional and behavioural problems may vary among adolescents from different sociodemographic groups. However, epidemiological evidence of changes in the incidence of adolescent psychopathology across the last decade is lacking. Insight into secular changes in emotional and behavioural problems for different sociodemographic groups is crucial for adequate policy focusing on adolescent mental health [7].

Evidence on time trends in the prevalence of emotional and behavioural problems among adolescents in Western countries, including The Netherlands, is inconsistent [1, 2, 11–21]. Several studies found an increase in the prevalence of emotional and behavioural problems among adolescents during recent decades (e.g., [2, 17, 18]), while other studies found stable or decreased levels (e.g., [11, 19–21]). These inconsistencies could be due to differences in study design, outcome measures, and study population. More importantly, a constraint in most studies is the use of only a few time points over a large time span.

Studies examining differences in time trends between different sociodemographic groups are scarce, which have focused mainly on differences between boys and girls and also showed inconsistent findings. The time trends for boys and girls differed with respect to specific emotional and behavioural problems and for self-reported and parent-reported questionnaires (e.g., [3, 14, 15, 17, 22]). Only a few studies examined differences for other sociodemographic factors; they found no evidence for differential time trends in emotional problems related to family composition [17] or for differential time trends in emotional and behavioural problems related to ethnic background, except for peer problems [15]. Ethnic differences in time trends were small between 2003 and 2005 with non-western adolescents reporting more peer problems, whereas no differential time trends related to ethnic background existed in 2007 and onwards. For educational level, only hyperactivity/inattention showed differential changes over time [15]. Differences in time trends between adolescents following vocational and academic educational levels were negligible in 2003, most pronounced in 2005 and small in 2007 and onwards, with academic students showing less hyperactivity.

To reliably examine time trends in adolescents’ emotional and behavioural problems, identical and repeated outcome measurements over a number of years in the same social and geographic population are needed [1, 13, 15, 17]. Studies meeting these criteria are rare. In the present study, we examined 10-year time trends in emotional and behavioural problems among 13–14-year-old multi-ethnic Dutch adolescents in Amsterdam at 1-year time points using the internationally validated Strengths and Difficulties Questionnaire (SDQ), and differences in these time trends between sociodemographic groups. We present mean scores to provide a general view of changes in social–emotional wellbeing in adolescents. As the SDQ is a screening measure, we particularly concentrate on adolescents with a relatively high (abnormal) score as an indicator of serious emotional and behavioural problems.

## Methods

### Sample and materials

In all primary and secondary schools in Amsterdam, there are obligatory general medical examinations of pupils. The consult consisted of a base set of physical examinations and a questionnaire which is given before the physical examination. The data discussed in this paper concern this questionnaire administered among the second-year students (13–14 years old) at secondary schools. Exceptions were students attending a number of conservative religious schools, private schools, international schools, and schools for special educational needs. This concerned around 175 of about 6000 students per school year. The questionnaire included the sociodemographic characteristics and the lifestyles and health profile of the students. The SDQ was part of this questionnaire, whereby the content and wording of the SDQ remained unchanged during the period of observation. The questionnaire is completed in class in an exam room set up, under supervision of a teacher and school nurse of the Public Health Service Amsterdam. To avoid socially desirable answers, the school nurse explained to the students that their answers were kept confidential and only known to the school nurse or possibly a physician. Students who have a score indicating a possible health risk were followed up during the subsequent physical examination by the school nurse or school physician. From school years 2004–2005 to 2009–2010, data were collected by a paper questionnaire; from school years 2010–2011 to 2013–2014, an electronic questionnaire was used. Before data collection, information letters were sent to parents and students. A passive informed consent procedure was used, so students and their parents could decide to not to complete the questionnaire. The response rate on the questionnaire was around 90% annually. The most common reason for non-response was illness of the student on the day which the questionnaire was given. All respondents were living in Amsterdam or nearby (e.g., Aalsmeer, Uithoorn, Ouder-Amstel, and Amstelveen), which is a predominantly urban area. The percentage of students screened fluctuates between years, mainly due to variations in the length of the questionnaire and the physical examination and changes in staffing levels. Before analysis, we examined whether the annual data were representative for Amsterdam by weighing the data by age, gender, ethnicity, and social status groups in a randomly selected year. The effects of weighing the SDQ data were negligible for the percentage adolescents with abnormal scores on the total difficulties score and subscales: ≤ 0.2%. We, therefore, assume that the students ascertained each year are representative of all students in Amsterdam. The data of all questionnaires are aggregated and anonymised and placed into a monitor to support the development of regional health policies and the elaboration of health promoting activities. The youth health monitor is registered at the Dutch Data Protection Authority. In the present study, only questions about emotional and behavioural problems and sociodemographic characteristics were used.

### Measures

#### Emotional and behavioural problems

Emotional and behavioural problems were assessed by the SDQ, which is a 25-item screening questionnaire that asks students to report on their behaviours and emotions in the past 6 months [23–25]. The items are distributed across five scales of five items each: emotional problems, conduct problems, hyperactivity/inattention, peer-relationship problems, and pro-social behaviour. Items are scored on a three-point Likert scale (‘not true’, ‘somewhat true’, ‘certainly true’, and scored 0–2). Examples of items are: ‘I have many fears, I am easily scared’, ‘I get a lot of headaches, stomach-aches or sickness’, and ‘I am restless, I cannot stay still for long’. Only the first four scales were included in this study as the fifth scale (pro-social behaviour) is not used in calculating the SDQ total score. Subscale scores were only computed for participants who completed at least three of the five items in each subscale, the item mean was used in the case of one or two missing items. In less than 1% of students, the SDQ total score could not be calculated because of too few questions answered. The subscales were each scored from 0 to 10. A total difficulties score was calculated as the sum of scores of the four subscales [23, 26]. To determine subgroups (normal versus abnormal) for both the total difficulties scale and subscales, the scores were dichotomized according to the cut-off points used by the Amsterdam Child Health Care Department (abnormal scores: total difficulties scale > 15, emotional problems > 5, conduct problems > 3, hyperactivity/inattention > 5, and peer-relationship problems > 3) and were the same for both genders. These cut-off points were based on statistical analyses (ROC analyses with elevated ASEBA scores as the criterion) and clinical practice (minimizing the chance to miss true cases) [27]. The previous studies showed a good validity and reliability of the SDQ self-report version in Dutch adolescents attending lower to higher educational levels [25, 28, 29]. The SDQ has been used throughout the world and translated into more than 80 languages [30, 31]. Nevertheless, further research is needed to confirm its use in cross-cultural comparisons [32, 33].

#### Sociodemographic characteristics

The sociodemographic characteristics gathered in the questionnaire were age, sex, educational level, ethnicity, and family composition.

Ethnicity was based on self-reported country of birth from both the student and his/her parents. In accordance with the definition used by Statistics Netherlands, we defined a student to be of non-Dutch ethnic background when at least one parent was foreign-born [34]. We categorized ethnicity into the five largest groups in the Netherlands: Dutch, Surinamese, Turkish, Moroccan, and other.

Family composition was assessed by asking with whom the student lived most days of the week: living together with father and mother, living together with father/mother and partner, co-parenting, single-parent family, or other living arrangement.

Education was divided in four categories according to the Dutch secondary school: the practical track of preparatory vocational secondary education (PT VMBO), preparing for manual jobs; the theoretical track of preparatory vocational secondary education (TT VMBO), preparing for administrative jobs; senior general secondary education (HAVO), preparing for professional (higher) education; and pre-university education (VWO), preparing for university. Many secondary schools offer mixed classes giving students 1 or 2 years to show at what level which they are capable of performing. For the present study, adolescents who attended second-year mixed-level education were categorized as ‘other’ [35].

### Statistical analysis

Data analysis was performed using IBM SPSS Statistics 21. Abnormal scores on the SDQ total difficulties scale and the four problem subscales were compared across the 10 years. We used logistic regression analysis with time as an independent variable to examine if the prevalence of abnormal scores on the total difficulties scale and subscales changed over time, and adjusted for age, sex, ethnicity, family composition, and educational level. During the observation period, the data collection mode changed from pencil and paper to electronic. We routinely corrected for this mode change by including a before and after change dummy variable in the analysis. We corrected the figures by subtracting the mode effect from the time trend after the mode change. This means that all percentages reported in this paper represent values according to the pencil and paper mode. We also examined mode effects on the time trends. Generalized linear models (GLM) analyses were performed to model time trends in SDQ mean scores across the years. We examined interactions between time and sex, ethnicity, family composition, and educational level to explore whether time trends of abnormal scores on the total difficulties scale and the four subscales varied between sociodemographic groups. As suggested by Stern and Smith [36], we present 95% confidence intervals—indicating the precision and potential range of our estimates—as well as precise *P* values, without reference to some arbitrary threshold. The smaller the *P* value, the stronger the evidence.

## Results

### Population characteristics

The number of respondents varied between 4844 in 2005–2006 and 6884 in 2013–2014. In total, 56,159 respondents were included in the analysis. In 2005–2006, the number of children screened was relatively small due to staff shortages as a result of reorganization. Other fluctuations were also observed, in all periods; however, we assume that the data were representative for students attending schools in Amsterdam. The mean age of the participants was 13.6 years (Table 1).

**Table 1:** Sociodemographic characteristics of 13–14-year-old adolescents participating in the youth health monitor between school years 2004–2005 and 2013–2014

	2004	2005	2006	2007	2008	2009	2010	2011	2012	2013	Total
Participants (*n*)	5465	4844	4921	5237	4637	5475	6202	6542	5952	6884	56,159
Age (years)											
Mean	13.7	13.7	13.6	13.6	13.5	13.6	13.6	13.5	13.7	13.6	13.6
SD	0.7	0.7	0.7	0.7	0.7	0.7	0.7	0.6	0.7	0.6	0.7
Sex (%)											
Boys	48.0	50.5	49.4	49.8	50.3	49.0	49.0	49.4	49.7	48.8	49.3
Girls	52.0	49.5	50.6	50.2	49.7	51.0	51.0	50.6	50.3	51.2	50.7
Ethnicity (%)^*^											
Dutch	35.1	39.0	36.4	35.9	38.3	38.3	39.9	38.7	39.0	39.3	38.1
Surinamese	16.3	11.3	13.1	14.9	12.0	14.1	12.6	11.3	11.6	11.6	12.8
Turkish	10.2	10.6	11.0	9.7	10.0	9.6	9.2	9.6	8.9	8.4	9.6
Moroccan	17.0	18.1	18.6	17.2	15.8	15.8	16.0	16.4	15.5	16.3	16.6
Other	21.5	21.1	21.0	22.4	23.8	22.2	22.3	24.0	25.1	24.3	22.9
Family composition (%)											
Father and mother	66.1	68.5	66.4	66.2	65.4	64.4	67.1	69.1	67.3	67.0	66.8
Parent and partner	4.0	3.3	4.7	4.8	4.6	4.9	3.9	3.2	3.6	5.0	4.2
Co-parenting	7.8	7.6	3.5	3.2	4.4	4.6	7.1	7.2	8.1	8.2	6.3
Single-parent family	19.6	18.5	24.1	24.1	24.1	23.3	20.4	19.1	19.5	18.2	20.9
Other	2.6	2.2	1.4	1.6	1.5	2.8	1.5	1.3	1.5	1.5	1.8
Educational level (%)											
Practical pre-vocational	27.0	25.6	26.4	26.1	23.5	22.8	19.5	19.6	21.3	19.7	22.8
Theoretical pre-vocational	21.6	20.7	20.3	20.9	21.1	19.7	21.4	20.6	19.5	18.5	20.4
Senior general secondary	13.5	16.5	11.4	11.0	12.5	12.1	11.8	12.5	14.5	13.6	12.9
Pre-university	20.7	23.4	22.0	25.7	24.5	25.7	27.8	28.2	27.7	27.6	25.6
Other	17.2	13.7	19.9	16.3	18.4	19.7	19.5	19.1	17.0	20.6	18.3

2004 means school year 2004–2005, et cetera

*Ethnic background of the second-year students at secondary schools in Amsterdam during observation period, Statistics Netherlands’ database, see ‘Online resource 1’

### Emotional and behavioural problems

One out of ten adolescents had an abnormal score on the total difficulties scale. More than 7% of the students had abnormal scores on emotional problems, 13.2% on conduct problems, 18.7% on hyperactivity/inattention, and 12.0% on peer-relationship problems. The mean score on the total difficulties scale was 9. The mean scores on the subscales were 2.1 for emotional problems, 1.9 for conduct problems, 3.4 for hyperactivity/inattention, and 1.6 for peer-relationship problems (Table 2).

**Table 2:** SDQ-measured percentages of abnormal scores, and mean scores of emotional and behavioural problems between 2004–2005 and 2013–2014 of 13–14-year-old adolescents participating in the youth health monitor, Amsterdam, The Netherlands

	2004	2005	2006	2007	2008	2009	2010	2011	2012	2013	Total
Abnormal scores (%)											
Total difficulties	9.8	8.5	8.7	9.6	9.1	10.6	10.9	10.9	11.2	11.4	10.2
Emotional problems	7.5	6.4	6.3	7.5	6.7	6.8	7.7	7.9	8.3	8.6	7.4
Conduct problems	13.9	13.1	12.9	13.4	13.0	13.8	13.2	13.4	12.1	13.5	13.2
Hyperactivity/inattention	15.9	16.2	16.6	18.0	17.1	17.8	19.4	20.6	21.1	21.8	18.7
Peer-relationship problems	14.4	13.0	10.5	10.9	10.9	10.3	13.7	12.6	11.9	11.5	12.0
Mean scores											
Total difficulties	9.0	8.8	8.8	9.0	8.8	9.0	9.2	9.2	9.2	9.3	9.0
Emotional problems	2.1	2.0	2.1	2.2	2.1	2.2	2.1	2.2	2.2	2.2	2.1
Conduct problems	1.9	1.8	1.9	1.9	1.8	1.9	1.8	1.8	1.8	1.9	1.9
Hyperactivity/inattention	3.2	3.2	3.3	3.4	3.3	3.4	3.5	3.6	3.6	3.6	3.4
Peer-relationship problems	1.7	1.7	1.5	1.6	1.5	1.5	1.7	1.7	1.6	1.6	1.6

2004 means school year 2004–2005, et cetera

### Time trends in emotional and behavioural problems

**Fig. 1:**
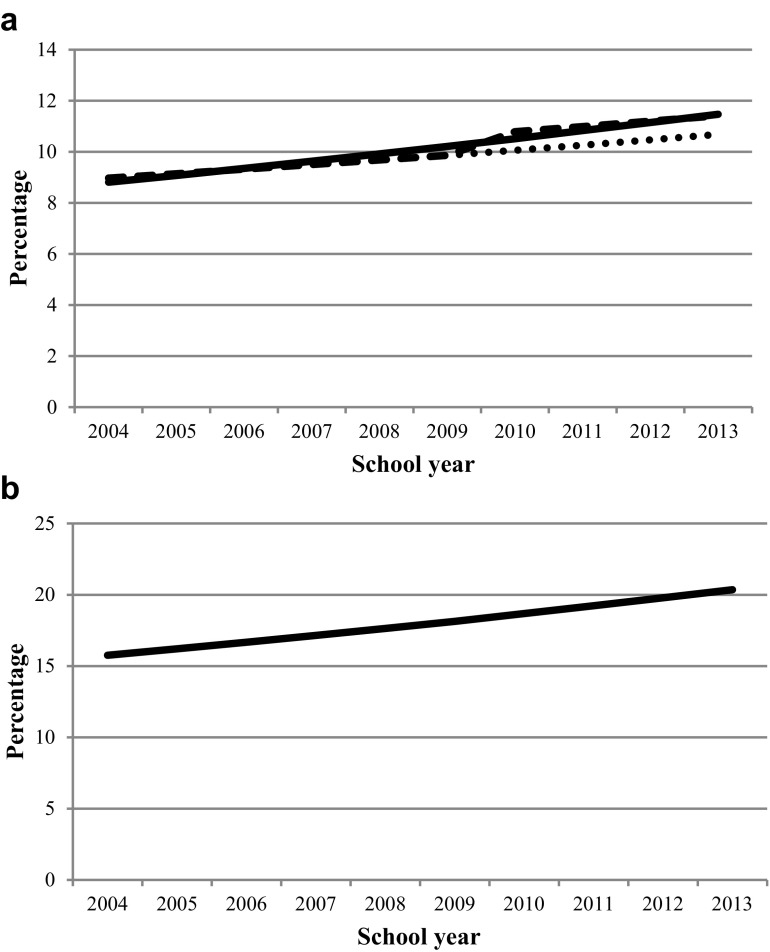
**a** Estimated percentage of 13–14 years old with abnormal scores on total difficulties, school year 2004–2005 until 2013–2014. Estimated percentage not considering change in data collection mode (line), estimated percentage considering change in data collection mode (dashed line), projected percentage considering change in data collection mode, e.g., the estimated percentages if the paper forms had been continued to be used (dotted line). **b** Projected percentage of 13–14 years old with abnormal scores on hyperactivity/inattention for school year 2004–2005 until 2013–2014

Table 3 presents the estimates of the time trends in the proportion abnormal scores and mean SDQ scores in the total difficulties scale. Prevalence of abnormal scores on the total difficulties scale increased (OR crude 1.02; 95% CI 1.00, 1.04: *P* = 0.03) during our period of observation of 10 years from 9.0% in 2004–2005 to 10.7% in 2013–2014, a relative increase of 18.9%, estimated on the basis of the regression analysis and adjusted for chance fluctuation and change in data collection mode. Figure 1a illustrates the adjustment for change in data collection mode (from pencil and paper to electronic). The dashed line in the figure shows the trend after correction for the mode change. The jump in the dashed line shows the effect of the mode change on the proportion of abnormal scores, with somewhat increased prevalence of abnormal scores (OR 1.08; 95% CI 0.97, 1.21: *P* = 0.15). We found no evidence for an effect of mode change on the time trend (OR 1.00; 95% CI 0.96, 1.04: *P* = 0.91). After controlling for the sociodemographic factors as previously mentioned, the observed increase in the total difficulties scale remained the same (OR adjusted 1.02; 95% CI 1.00, 1.04: *P* = 0.02).

**Table 3:** Ten-year time trends in SDQ-measured emotional and behavioural problems (abnormal scores and mean scores) among 13–14-year-old adolescents participating in the youth health monitor between school years 2004–2005 and 2013–2014, Amsterdam, The Netherlands

	OR Crude^a^	95% CI	*P*	OR Adjusted^b^	95% CI	*P*
Abnormal scores (results of logistic regression analysis)						
Total difficulties	1.02	1.00, 1.04	0.028	1.02	1.00, 1.04	0.022
Emotional problems	1.01	0.99, 1.03	0.468	1.01	0.98, 1.03	0.619
Conduct problems	1.00	0.98, 1.02	0.851	1.00	0.99, 1.02	0.504
Hyperactivity/inattention	1.03	1.02, 1.05	0.000	1.03	1.02, 1.05	0.000
Peer-relationship problems	0.93	0.91, 0,95	0.000	0.94	0.92, 0.95	0.000
Mean scores (results of generalized linear models analysis)						
Total difficulties	0.01	− 0.02, 0.03	0.222	0.01	− 0.14, 0.04	0.331
Emotional problems	0.02	0.01, 0.03	0.001	0.02	0.01, 0.03	0.002
Conduct problems	− 0.01	− 0,02, − 0.00	0.032	− 0.01	− 0.01, 0.00	0.239
Hyperactivity/inattention	0.04	0.03, 0.06	0.000	0.04	0.03, 0.05	0.000
Peer-relationship problems	− 0.05	− 0.06, − 0.04	0.000	− 0.04	− 0.05, − 0.03	0.000

*95% CI* 95% confidence interval

^a^Changes per annum, adjusted for change in data collection mode

^b^Changes per annum, adjusted for change in data collection mode, age, sex, ethnicity, family composition, and educational level

Prevalence of abnormal scores on hyperactivity/inattention increased from 15.8% in 2004–2005 to 20.4% in 2013–2014, a relative increase of 29.2% (Fig. 1b, OR crude 1.03; 95% CI 1.02, 1.05: *P* = 0.00; OR adjusted 1.03; 95% CI 1.02, 1.05: *P* = 0.00), while peer-relationships problems decreased (OR adjusted 0.94; 95% CI 0.92, 0.95: *P* = 0.00). Emotional problems (OR adjusted 1.01; 95% CI 0.98, 1.03: *P* = 0.62) and conduct problems (OR adjusted 1.00; 95% CI 0.99, 1.02: *P* = 0.50) hardly changed over time. Effects of the mode change from pencil and paper to electronic were inconsistent: increases in emotional problems (OR 1.14; 95% CI 1.01, 1.30: *P* = 0.04), hyperactivity (OR 1.09; 95% CI 1.00, 1.18: *P* = 0.06) and peer-relationship problems (OR 1.55; 95% CI 1.40, 1.71: *P* = 0.00), and a slight decrease in conduct problems (OR 0.98; 95% CI 0.89, 1.08: *P* = 0.74). The effect of the mode change on the trends in the subscales was, in general, small (OR 1.05; 95% CI 1.00, 1.10: *P* = 0.05; OR 1.02; 95% CI 0.99, 1.05: *P* = 0.24: OR 1.01; 95% CI 0.97, 1.05: *P* = 0.71; OR 1.00; 95% CI 0.96, 1.04: *P* = 0.88, respectively).

### Time trends in emotional and behavioural problems according to sex

**Fig. 2:**
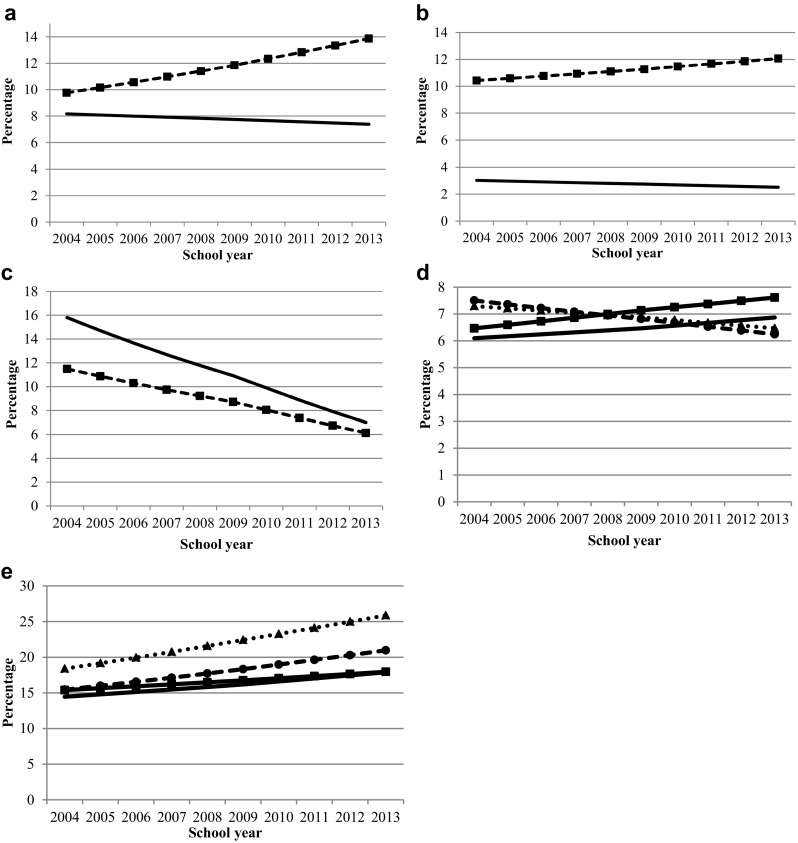
**a** Projected percentage of 13–14 years old with abnormal scores on total difficulties, stratified by sex ( boys,  girls) for school year 2004–2005 until 2013–2014. **b** Projected percentage of 13–14 years old with abnormal scores on emotional problems, stratified by sex ( boys,  girls) for school year 2004–2005 until 2013–2014. **c** Projected percentage of 13–14 years old with abnormal scores on peer-relationship problems, stratified by sex ( boys,  girls) for school year 2004–2005 until 2013–2014. **d** Projected percentage of 13–14 years old with abnormal scores on emotional problems, stratified by education levels [ pre-university education (VWO),  senior general secondary education (HAVO),  theoretical track of preparatory vocational secondary education (TT VMBO),  practical track of preparatory vocational secondary education (PT VMBO)] for school years 2004–2005 until 2013–2014.** e** Projected percentage of 13–14 years old with abnormal scores on hyperactivity/inattention, stratified by education levels (VWO, HAVO,  TT VMBO, PT VMBO) for school year 2004–2005 until 2013–2014

We found some evidence for different time trends in boys and girls for the total difficulties scale (OR adjusted 0.96; 95% CI 0.94, 0.98: *P* = 0.00), emotional problems (OR adjusted 0.97; 95% CI 0.94, 0.99: *P* = 0.01), and peer-relationship problems (OR adjusted 0.96; 95% CI 0.95, 0.98: *P* = 0.00) but not very different for conduct problems (OR adjusted 0.99; 95% CI 0.98, 1.01: *P* = 0.47) and hyperactivity (OR adjusted 0.99; 95% CI 0.98, 1.01: *P* = 0.36). Girls had an increasing time trend in abnormal scores on total difficulties (OR adjusted 1.05; 95% CI 1.02, 1.07: *P* = 0.00) and emotional problems (OR adjusted 1.02; 95% CI 0.99, 1.04: *P* = 0.25), while boys hardly changed (OR adjusted 0.99; 95% CI 0.96, 1.02: *P* = 0.58 and OR adjusted 0.98; 95% CI 0.93, 1.03: *P* = 0.32, respectively). For both boys and girls, abnormal scores on peer-relationship problems decreased (OR adjusted 0.93; 95% CI 0.90, 0.95: *P* = 0.00; and OR adjusted 0.95; 95% CI 0.93, 0.98: *P* = 0.00; respectively) (Fig. 2a–c).

### Time trends in emotional and behavioural problems according to educational level

We found evidence for differences in time trends between different educational levels in emotional problems (Chi-sq 26.1; *df* 4; *P* = 0.00) and hyperactivity/inattention (Chi-sq 16.0; *df* 4; *P* = 0.00) and small differences for the total difficulties scale (Chi-sq 8.2; *df* 4; *P* = 0.09), peer-relationship problems (Chi-sq 2.8; *df* 4; *P* = 0.60), and conduct problems (Chi-sq 1.6; *df* 4; *P* = 0.80). For abnormal scores on emotional problems, we found increasing time trends for adolescents attending the practical track of preparatory vocational secondary education (OR adjusted 1.02; 95% CI 0.98, 1.07: *P* = 0.37) and pre-university education (OR adjusted 1.01; 95% CI 0.97, 1.06: *P* = 0.58) and decreasing time trends for adolescents attending the theoretical track of preparatory vocational secondary education (OR adjusted 0.98; 95% CI 0.94, 1.03: *P* = 0.40) and senior general secondary education (OR adjusted 0.99; 95% CI 0.93, 1.05: *P* = 0.67). For all educational levels, we found increasing time trends for abnormal scores on hyperactivity/inattention. Adolescents attending the theoretical track of preparatory vocational secondary education (OR adjusted 1.04; 95% CI 1.01, 1.08: *P* = 0.01) and senior general secondary education (OR adjusted 1.05; 95% CI 1.01, 1.09: *P* = 0.01) had the largest time trends for abnormal scores on hyperactivity/inattention (Fig. 2d, e).

## Discussion

Overall, the time trends in emotional and behavioural problems among adolescents in Amsterdam varied according to type of problem. The total difficulties and hyperactivity/inattention scores increased over the 10-year time period. The number of adolescents with an abnormal total difficulties score increased with 18.9% over this period and the number of adolescents with an abnormal score on hyperactivity/inattention with 29.2%. We found differences in time trends in emotional and behavioural problems between boys and girls (i.e., total difficulties, emotional problems, and peer-relationship problems) and for educational level (i.e., emotional problems and hyperactivity/inattention).

### Changes in emotional and behavioural problems

The percentage of adolescents with abnormal scores on total difficulties increased as did the mean of the total difficulties scores. For 2004–2005, about 9.0% of pupils were at more serious levels of emotional and behavioural problems which increased to 10.7% in 2013–2014. This increase was mainly due to an increase in hyperactivity/inattention problems and to a lesser extent to emotional problems. One explanation is that adolescents became more willing to admit having emotional and behavioural problems over time. It is also possible that the above-mentioned societal changes caused the increase in emotional and behavioural problems. For example, increased pressure on adolescents from parents and teachers to perform well at school might induce stress. The rise in social media use puts pressure on adolescents due to the fear of missing out and being left out. Furthermore, cyber bullying, a relatively new phenomenon, can negatively influence adolescent’s emotional and social health [2, 3, 7, 37, 38]. To decide which interventions or preventive measures are needed, the causes behind the increasing trends in emotional and behavioural problems must be identified.

### Increase in hyperactivity/inattention

The observed increase in self-reported hyperactivity/inattention problems may be due to increased awareness. Increased awareness could enhance diagnoses and subsequent treatment for adolescents with hyperactivity/inattention. Several studies have, indeed, reported an increase in hyperactivity/inattention diagnosis and treatment among adolescents [39–41]. It is also possible that a true increase in hyperactivity/inattention symptoms has occurred. Several environmental factors might play a role in this trend: increased air and noise pollution, which might have a negative impact on the neuropsychological development of children; increasing use of social media including exposure to quickly delivered media messages of short duration; and excessive exposure to screen media and video games, which exacerbate attention problems [40, 42, 43].

### Gender differences

The findings emphasize that we must be careful to state that emotional and behavioural problems increased in adolescents in general. For example, a clear increase in total difficulties and emotional problems was found for girls but not for boys. This finding is in line with a study of West and Sweeting [3] in 15 years old. They found that emotional problems remained quite stable for boys, but the rates for girls almost doubled between 1987 and 1999. Moreover, recent research has shown that girls are not necessarily more willing than boys to report emotional problems [44]. It is possible that societal changes (e.g., worries about your looks, cyber bullying, and doing well at school) have a greater impact on adolescent girls than boys [3, 7, 45]. Another possible explanation is that recent generations of girls have experienced earlier onset of puberty, which has been associated with higher risk of depression and low self-esteem [46–48].

### Strengths and limitations

A major strength is that, to our knowledge, the present study is the first large-scale study examining time trends in emotional and behavioural problems, using the same validated outcome measure, study design, and population annually. Another major strength is the high participation rate. We, therefore, assume that the students who participated annually were representative of all students in Amsterdam for the respective years. Most Amsterdam school children also lived in Amsterdam and vice versa. Our study is, therefore, likely representative for emotional and behavioural problems in adolescents in an urban area. One limitation is our reliance on self-report instead of a multi informant approach. However, research showed that each category of informant is valuable and unique for identifying emotional and behavioural problems in adolescents. Furthermore, that internalizing problems are less apparent to parents and teachers [49, 50]. A second possible limitation is whether these trends reflect changes in actual prevalence of emotional and behavioural problems, or whether adolescents are more frank to report about emotional and behavioural problems than in the past. Our findings do not suggest a greater likelihood of disclosing problems, given that increases were restricted to certain problems and were not consistently across the different sociodemographic groups, so more willingness to report emotional and behavioural problems in general seems unlikely. We used the same cut-off points for both genders, since this is also done in screening practice. Moreover, using gender specific cut-off points would lead to smaller or even no gender differences in the prevalence of abnormal scores despite actual higher scores among girls or boys. It is, however, unlikely that this influenced gender differences in time trends. Finally, our study was confidential (non-anonymous). However, a previous Dutch study among adolescents in a similar setting has shown that that there was no significant difference in results on the SDQ between confidentially and anonymous collected data [51].

As suggested by Stern and Smith [36], we present 95% confidence intervals—indicating the precision and potential range of our estimates—as well as precise *P* values, without reference to some arbitrary threshold. In addition, we view our paper as exploratory in the context of developing knowledge on trends in adolescent mental health, and invite researchers to replicate our study in other samples.

A limitation of our data is that the mode of data collection changed from pencil and paper to electronic means. This may have influenced adolescent’s responses. We, indeed, found some effect of mode change in the level of the total difficulties and subscales, and very limited effects in time trends. We adjusted the analyses for mode changes in the level. We based this paper on the paper and pencil mode after the correction for mode effects. However, based on our data, we cannot state which mode is more accurate.

### Implications for further research

Although our explanations for the observed differences in time trends are based on recent literature, the above-mentioned potential causes are fairly speculative. To really understand why emotional and behavioural problems have increased over time in some sociodemographic groups and not in others, we need to combine social sciences and epidemiology, and look at both qualitative and quantitative methodologies. Finally, we cannot totally exclude that potential confounding underlies our results.

### Implications for health policy

Increases in the prevalence of emotional and behavioural problems leads to increases in costs. Public health services, mental health services, and schools have to deal with increasing numbers of adolescents at high risk for emotional and behavioural problems. Furthermore, high rates of emotional and behavioural problems during adolescence are risk factors for psychopathology and long-term work disability in young adulthood [8–10]. To address this situation, further time trend studies among diverse subgroups are needed to confirm our observations and to enable policy makers to make well-supported, long-term decisions about prevention and care.

## Electronic supplementary material

Below is the link to the electronic supplementary material.

Supplementary material 1 (PDF 75 KB)
