# Supplementary material for: Sociodemographic differences in 10-year time trends of emotional and behavioural problems among adolescents attending secondary schools in Amsterdam, The Netherlands
Source: Eur Child Adolesc Psychiatry. 2018 Apr 26;27(12):1621–31. doi: 10.1007/s00787-018-1157-5 (PMC6245132; doi:10.1007/s00787-018-1157-5)
Supplement: Supplementary file 1 — Supplementary material 1 (PDF 75 KB) [file 787_2018_1157_MOESM1_ESM.pdf]

Journal: European Child & Adolescent Psychiatry.

Title: Sociodemographic differences in ten-year time trends of emotional and behavioural problems among adolescents attending secondary schools in Amsterdam, the Netherlands.

Cornelia L. van Vuuren[1,2], Daan G. Uitenbroek[1], Marcel F. van der Wal[1], Mai J.M. Chinapaw[2]

[1]Department of Epidemiology, Health Promotion and Healthcare Innovation, Public Health Service (GGD) Amsterdam, PO Box 2200, 1000 CE Amsterdam, The Netherlands

[2] Department of Public and Occupational Health, Amsterdam Public Health research institute, VU University Medical Center, Amsterdam, Van der Boechorststraat 7, 1081 BT Amsterdam, the Netherlands

Correspondence to:
Cornelia Leontine van Vuuren,
E-mail: lvvuuren@ggd.amsterdam.nl

Online resource 1: Ethnic background of second-year students at secondary schools in Amsterdam between school years 2004-2005 and 2013-2014 from the Statistics Netherlands' database.

	**2004**	**2005**	**2006**	**2007**	**2008**	**2009**	**2010**	**2011**	**2012**	**2013**
**Ethnicity (%)**										
Dutch	37.1	36.9	38.0	37.3	38.5	38.5	38.3	37.5	38.8	38.4
Surinamese	15.0	13.7	13.0	13.4	12.5	12.6	11.7	10.8	10.4	10.6
Turkish	9.6	9.8	9.6	9.3	8.7	9.1	9.1	9.4	8.6	8.7
Moroccan	17.3	17.4	16.4	16.3	15.9	15.7	16.5	16.2	15.6	16.3
Other	21.0	22.2	23.0	23.7	24.5	24.1	24.4	26.1	26.6	26.0

2004 means school year 2004-2005.
